# A Microencapsulation Method for Delivering Tetrodotoxin to Bivalves to Investigate Uptake and Accumulation

**DOI:** 10.3390/md19010033

**Published:** 2021-01-13

**Authors:** Laura Biessy, Kirsty F. Smith, Susanna A. Wood, Annabel Tidy, Roel van Ginkel, Joel R. D. Bowater, Ian Hawes

**Affiliations:** 1 Cawthron Institute, Coastal and Freshwater, Nelson 7010, New Zealand; kirsty.smith@cawthron.org.nz (K.F.S.); susie.wood@cawthron.org.nz (S.A.W.); roel.vanginkel@cawthron.org.nz (R.v.G.); joel.bowater@cawthron.org.nz (J.R.D.B.); 2 Department of Biological Sciences, University of Waikato, Hamilton 3216, New Zealand; ian.hawes@waikato.ac.nz; 3 New Zealand Food Safety Science & Research Centre, Palmerston North 4442, New Zealand; 4 School of Geography, Earth and Environmental Sciences, University of Birmingham, Birmingham B15 2TT, UK; a.tidy@bham.ac.uk

**Keywords:** bioaccumulation, feeding experiment, humic acid, marine toxin, shellfish

## Abstract

Most marine biotoxins are produced by microalgae. The neurotoxin tetrodotoxin (TTX) has been reported in many seafood species worldwide but its source is unknown, making accumulation and depuration studies in shellfish difficult. Tetrodotoxin is a water-soluble toxin and cannot be directly ingested by shellfish. In the present study, a method was developed which involved binding TTX to solid particles of humic acid and encapsulating them in agar-gelatin capsules. A controlled quantity of TTX-containing microcapsules (size range 20–280 μm) was fed to *Paphies australis*, a bivalve known to accumulate TTX in the wild. The TTX-containing microcapsules were fed to *P. australis* every second day for 13 days. Ten *P. australis* (including five controls fed non-toxic microalgae) were harvested after 7 days and ten after 13 days. *Paphies australis* accumulated TTX, reaching concentrations of up to 103 µg kg^−1^ by day 13, exceeding the European Food Safety Authority recommended concentration of 44 μg kg^−1^ in shellfish. This novel method will allow future studies to explore the effects, accumulation and depuration rates of TTX in different animals and document how it is transferred through food webs.

## 1. Introduction

Shellfish are a rich source of protein, essential minerals, vitamins and are an important food source worldwide [1]. However, bivalves filter large volumes of water and can concentrate contaminants including bacterial pathogens and phycotoxins [2]. With over 66 million tonnes of shellfish now consumed by humans annually, the risk of poisoning through contaminated seafood is an increasing public health concern [3,4]. With regards to phycotoxins (biotoxins produced by microalgae), the risk of poisoning increases exponentially during harmful algal blooms (HABs), when microalgal populations form dense concentrations of cells and sometimes visible water discolouration [5]. HABs have negative environmental impacts and can cause mass mortalities of fish, birds and marine mammals, and human illness [1], when they produce biotoxins that contaminate seafood through biomagnification up the food web [6]. About 300 marine microalgal species are known to produce biotoxins and more than 100 of these can cause intoxication or even death in humans and animals [6].

The microalgal species responsible for producing most of the known biotoxins have been identified. For example, in the marine environment, saxitoxin (STX), a potent neurotoxin regularly present in edible shellfish and responsible for paralytic shellfish poisoning, is produced by dinoflagellates species from the genera *Alexandrium, Gymnodinium, Centrodinium* and *Pyrodinium* [7,8,9,10]. Saxitoxin is also produced by freshwater cyanobacteria, the most common genera being *Anabaena*, *Aphanizomenon*, *Cylindrospermopsis*, *Lyngbya* and *Planktothrix* [11,12,13]. The fate of microalgal biotoxins in marine and freshwater organisms has been evaluated in a number of species. For example, green-lipped mussels *Perna viridis* were exposed to STX-producing dinoflagellate *Alexandrium fundyense* that were then fed to black sea bream *Acanthopagrus schlegeli* *and* the accumulation, distribution, transformation, and elimination of STX in specific organs were evaluated [14]. The hepatopancreas in the mussels and the viscera in the fish accumulated most of the STX. Differences in uptake, distribution, and elimination of STX were observed between mussels and fish, and this may influence the trophic transfer of microalgal toxins in marine organisms. Pereira et al. [15] evaluated the accumulation and depuration of STX by a freshwater mussel *Anodonta cygnea* exposed to the STX-producing cyanobacterium *Cuspidothrix* (Aphanizomenon) issatschenkoi, and concluded that when removing/metabolizing 40% of the daily toxin consumed, the feeding behaviour of the mussels was affected (i.e., erratic feeding patterns and lower clearance rate of the toxic species).

While the sources and pathways of many marine biotoxins are well known, the origin of tetrodotoxin (TTX), a widespread and potent neurotoxin naturally occurring in organisms from marine, freshwater and terrestrial environments, remains uncertain [16]. There is contradictory evidence regarding whether the source of TTX is exogenous or endogenous, and the pathways and mechanisms through which TTX is incorporated in the food web are unknown [17]. The wide distribution of TTX in many genetically unrelated species suggests that the toxin comes from an exogenous source such as accumulation through diet or symbiotic bacteria [18,19] with reports in the literature of at least 150 TTX-producing bacterial strains [20]. However, there is also evidence for an endogenous source in terrestrial species such as frogs or newts [21,22]. The neurotoxin is responsible for 30–50 global human intoxications every year and has the highest fatality rate of all marine biotoxins, mainly from pufferfish (e.g., fugu) consumption [23]. Tetrodotoxin also occurs in marine shellfish and has been reported in 21 species of bivalves and edible gastropods from ten countries since the 1980s [24]. The risk of TTX intoxication to humans via shellfish cannot be ignored due to the high and increasing amounts consumed worldwide. Understanding the accumulation of TTX in the marine food web will help evaluate and reduce this risk. Without a known producer, studies which explore TTX accumulation in bivalves are very challenging. To date, there have been no feeding studies in bivalves, but feeding studies involving other organisms such as the sea-slug *Pleurobranchaea maculata* [25] and the pufferfish *Takifugu niphobles* [26] suggested that TTX is accumulated via the diet.

A possible option for administrating toxins to bivalves is through microencapsulation. This involves the encapsulation of a solid within a thin protective coating, creating small particles [27]. This technology has been readily used within food and pharmaceutical industries as it allows a core material to be completely isolated from the external environment [28]. The technique has recently been used to provide sustained and controlled release of bioactives in aquaculture [29]. Microencapsulation has been used to feed live microalgae to the oyster *Crassostrea gigas* [27] but the present study is the first which uses the encapsulation of a pure toxin which is then fed to shellfish. Tetrodotoxin is a relatively small, water-soluble molecule [30], making it impossible to directly encapsulate TTX without it being bound to a solid first.

This study aimed to (1) develop a method to encapsulate TTX into a food suitable for bivalves and (2) feed known amounts of TTX to a bivalve species to determine accumulation. This technique would allow future studies to investigate the effects, accumulation and depuration rates of the neurotoxin in different animals, as well as its bioaccumulation in higher organisms via feeding of contaminated shellfish. This method could then be applied to other biotoxins where the source is unknown, isolation of the causative organisms is challenging or production of the biotoxin is not stable. In this experiment, TTX bound to humic acid was encapsulated in an agar-gelatin solution. A known quantity of the TTX microcapsules were fed to *Paphies australis*, an endemic New Zealand clam that has been shown to accumulate high TTX concentrations in the wild [31,32].

## 2. Results

### 2.1. Microscopic Characterization of Microcapsules

To develop the encapsulation method, microalgae that could be observed under the microscope were first used to determine if cells could be encapsulated and if the capsules were impermeable.

**Figure 1:**
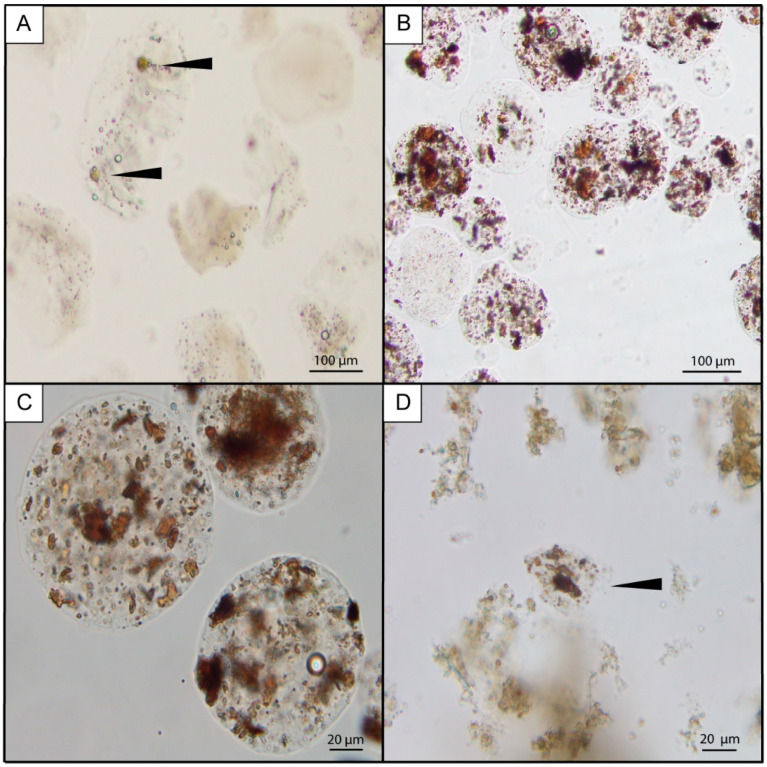
Agar-gelatin microcapsules containing *Alexandrium minutum* cells ((**A**); black arrows) and humic acid (the brown solids; (**B**–**D**)). The black arrow in (**D**) shows a partially digested capsule containing humic acid that was found in the digestive gland of a *Paphies australis*.

The microscopic analysis showed the microcapsules were intact and approximately spherical (Figure 1). The encapsulation of the live *Alexandrium minutum* cells was successful (Figure 1A) and the micro-algae survived the high temperatures of the encapsulation and stayed in the capsules until they disintegrated (42 h). The microscopic analysis also showed that the humic acid was encapsulated (Figure 1B,C). The smallest capsules agglutinated to each other but were easily disrupted with gentle agitation. The capsules stayed intact for a minimum of 34 h, their diameters ranged from 20 to 280 µm and >85% contained humic acid particles. Each fresh batch of capsules (made just prior to feeding) varied between 18,000 and 33,000 capsules per mL and ca. 75 mL of concentrated capsule solution was produced, containing ca. 506 µg TTX per L^−1^. On average, each *P. australis* filtered the 100 mL of solution (93 mL of microalgae with 7 mL of TTX capsules) within 4 h. Capsules containing humic acid particles were found partially digested in the digestive glands of *P. australis* (Figure 1D), showing that the clams were capable of filtering and digesting the agar-gelatin capsules.

### 2.2. Tetrodotoxin Encapsulation

The next step was to validate that TTX was binding to humic acid before adding it to the capsules. The results showed that ca. 70% of the TTX was bound to the humic acid and that most (ca. 80%) of the bound TTX was released after addition of the formic acid to break the ionic bond between TTX and the humic acid, the same way as it would in the acidic digestive glands of *P. australis* (Table 1).

### 2.3. Feeding Experiment

**Figure 2:**
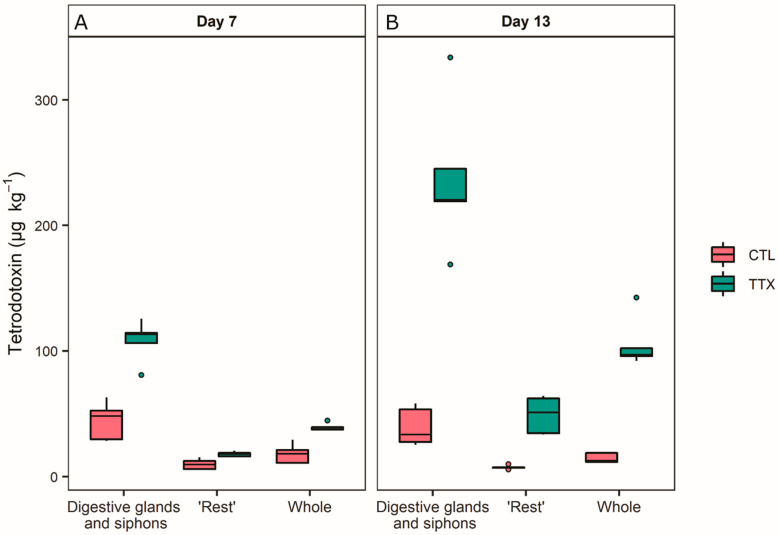
Tetrodotoxin (TTX) concentrations in experimental controls (CTL) and treatment samples (TTX) for the two tissue types (combined digestive gland and siphon, and everything else “rest”) and “whole” *Paphies australis* (*n* = 5) individuals after being fed controlled amounts of TTX every three days for 7 (**A**) and 13 days (**B**). The solid black line shows median, box shows 1st and 3rd quartiles, whiskers extend to the last data point within 1.5 times the inter-quartile range. Dots outside the whiskers are considered as outliers.

The culture of the microalga *Isochrysis gabana* did not contain any TTX. The environmental controls (wild *P. australis* harvested from Delaware Bay, Nelson, New Zealand) tested positive for TTX with an average of 15 µg kg^−1^. The TTX concentrations in the experimental controls remained stable during the feeding experiment (average of 17 and 15 µg kg^−1^ in whole *P^.^ australis* after 7 and 13 days respectively). TTX concentrations in the different samples of the experimental controls also remained constant with the digestive glands and siphons (DGS) containing an average of 44 µg kg^−1^ after 7 days and 39 µg kg^−1^ after 13 days and the “rest” of the organs containing 9.8 µg kg^−1^ after 7 days, and 7.5 µg kg^−1^ after 13 days (Figure 2).

Tetrodotoxin concentrations were significantly higher in the DGS of *P. australis* fed TTX for 7 days (average of 108 µg kg^−1^, *p* = 0.001) and 13 days (average of 238 µg kg^−1^, *p* < 0.0001), compared to the experimental control organisms fed microalgae only (average of 44 µg kg^−1^). The TTX concentration was also significantly higher in the “rest” samples compared to the controls (average of 9 µg kg^−1^) after 7 days of feeding (average of 18 µg kg^−1^, *p* = 0.026) and after 13 days (average of 49 µg kg^−1^, *p* < 0.0001). The *P. australis* accumulated more TTX over time, the concentration was significantly higher in DGS and in the “rest” samples at day 13 compared to day 7 (*p* = 0.0136 and *p* = 0.001, respectively; Figure 2). TTX concentrations in whole *P. australis* (calculated by summing results from DGS and rest samples on a pro-rata weight) reached 39.2 µg kg^−1^ after 7 days and 105.9 µg kg^−1^ after 13 days.

A mixed-effect linear model showed that there was no significant difference in the control groups from the DGS and the “rest” groups (*p* = 0.999 and *p* = 0.993, respectively) between days 7 and 13 (Table 2). The interactions between days, organs and treatment (TTX versus experimental controls) were not significant. There were significant differences in the control treatments between the DGS and “rest” sample types from both sampling days (*p* < 0.0001 for days 7 and 13) and there were significant differences between the two sample types and controls (*p* < 0.0001) and at each day (*p* < 0.0001).

Based on the amount of TTX provided at each feeding (ca. 506 µg L^−1^ in the capsule solution), the *P. australis* accumulated on average 0.5% of the TTX provided for the first 7 days (0.6% in the DGS and 0.32% in the “rest”) and 0.98% on average after 13 days (0.88% in the DGS and 0.42% in the “rest”).

## 3. Discussion

The overarching aim of this study was to develop a method to encapsulate TTX in a way that made it possible to feed it in a controlled experiment to bivalves. Achieving this would allow studies on accumulation rates and add to evidence that TTX might be obtained through the food web. In this study, we used the clams *P. australis*, which are endemic to New Zealand and have been shown to accumulate TTX to reasonably high levels in the environment as our test organisms [32].

An initial concern when generating the microcapsules was that the wild *P. australis* had never been exposed to artificial food. The microcapsules containing TTX needed to be within the size range of particles that the organisms were able to ingest. Most bivalves filter particles greater than 5 µm in diameter with nearly 100% efficiency [33] and several studies reported an efficient uptake of particles with diameters of 200 to 300 μm in bivalves [27,34]. The microcapsules produced in the present study were within this size range (20 to 280 µm). Our observations supported the posit that the microcapsules were of a digestible size as the capsule-microalgae solution was filtered and the water was clear within 4 h and microscopic analysis showed capsules present in the digestive glands of *P. australis*.

The encapsulation process used in our study did not destroy or degrade the TTX as evidenced by the high concentration remaining in the final capsule solution. Additionally, the agar, gelatin and humic acid were not detrimental to *P. australis* survival or feeding, as all the individuals stayed healthy for the duration of the experiment. The advantage of using an agar-gelatin capsule is that it is stable (the capsules stayed intact for at least 34 h) and impermeable, but easily dissolves when placed in a slightly acidic medium (i.e., the digestive system of bivalves). The use of humic acid as a solid binding agent was an important adaptation which allowed the water-soluble TTX to be contained inside a microcapsule instead of leaking out into the water.

Once the encapsulation method was developed and the ingestion of TTX-filled capsules by *P. australis* was confirmed, the second aim of this study was to feed them a controlled amount of TTX and investigate its accumulation rate. TTX has been detected in all *P. australis* tested in previous studies [31,32,35] and we anticipated to detect the low concentrations of TTX (average of 15 µg kg^−1^) found in the “environmental control” *P. australis*. The amounts detected after both seven (3 feedings) and 13 days (5 feedings) were significantly higher than this and were on par with concentrations recently detected in wild *P. australis* in New Zealand [35,36]. An exception to this was the detection of very high concentrations (800 µg kg^−1^, i.e., 80-fold higher than detected in other studies) of TTX in 2014 [32]. One hypothesis could be that the *P. australis* in that study had consumed a highly toxic source such as eggs or larvae from the sea-slug *Pleurobranchaea maculata* or flatworm *Stylochoplana* sp. that have been shown to contain extremely high levels of TTX [37].

The concentrations measured in whole *P. australis* after only 13 days of feeding (103 µg kg^−1^) were well above the European Food Safety Authority recommendation concentration of 44 μg kg^−1^ for TTX in shellfish without seeing adverse effects in human health [38]. This result shows that monitoring TTX concentrations in edible bivalves is important and should be established in areas or countries where shellfish have been shown to contain high TTX concentrations and are regularly harvested for consumption. Food-borne TTX is a realistic scenario as previous studies have shown that its source in bivalves is likely exogenous, with reports of bacterial species such as *Vibrio* and *Pseudomonas* [16,20] or cyanobacteria, especially picocyanobacteria [36,39] suggested as potential producers. If one of these species is confirmed as the producer, the risks of TTX in edible seafood are likely to increase with climate change as both cyanobacteria and bacteria thrive at warmer temperatures [40,41]. This study shows that *P. australis* can accumulate TTX from an external source and strengthens the hypothesis that bivalves accumulate TTX from their diet.

The method developed in this study can now be used to feed a range of bivalve species to determine if they can also accumulate TTX to concentrations above the recommended threshold. In addition to accumulation rates, depuration could also be studied, which will add valuable information for the management of commercial species (i.e., blue mussel, oysters) that have been shown to accumulate TTX in several countries [24]. Investigating TTX accumulation in a wide range of bivalves will also assist in understanding the mechanisms of TTX accumulation, such as the potential presence of a TTX-binding protein. Lastly, this method will also pave the way for further studies that explore TTX accumulation through the food web. For example, once the shellfish have accumulated the TTX, these could be fed to higher trophic organisms in the food web (e.g., fish). Similar studies have previously been undertaken, for example, Oikawa et al. (2005) investigated the accumulation and depuration rates of STX in crabs that were fed toxic mussels after being exposed to toxic cyanobacteria [42].

We estimated that only 0.5–1% of the TTX administered was accumulated by *P. australis*. The low accumulation relative to the amount filtered could be due to some TTX molecules breaking down with the acid from the *P. australis* digestive system. Unfortunately, we were unable to test the amount of TTX in the faeces, so the quantity of TTX directly excreted is unknown. It is also possible that *P. australis* are only capable of accumulating a certain amount of TTX. It has been hypothesised that these bivalves contain unique TTX-binding proteins similar to those found in pufferfish and crabs [31,43,44] that allow them to store the toxin in certain organs. It is possible that the TTX-binding protein could become saturated when exposed to a high amount of the toxin in a short amount of time. The low accumulation could also be due to the 20% of TTX remaining bound to the humic acid and not being absorbed by the bivalves once ingested. Despite the low accumulation rate of TTX observed in this study, the fact that *P. australis* are slow detoxifiers/depurators for TTX [31], suggests that for this species to reach similar concentrations in the wild, the producer would either be present most of the time but in low quantities, or only present occasionally but in higher concentrations. The amount of TTX filtered in the wild could thus be very high for a brief period and the *P. australis* retains the toxin for a very long time. Additionally, the bivalve species may accumulate a TTX precursor molecule and biosynthesize it to TTX, making the TTX producer very difficult to find.

In this study, the siphons and the digestive glands accumulated more TTX than the remaining tissue, corroborating with previous studies that reported these organs accumulated the highest amount of TTX in the wild [45]. The “rest” group did not show an increase in TTX accumulation after 7 days of feeding but did increase after 13 days, suggesting that the toxin is migrating between the clam’s organs as it was previously indicated [31]. Studies have found reduced predation on shellfish with high STX concentrations [46,47], suggesting that the concentrated levels of TTX within *P. australis*’ siphons may be to protect the vulnerable organ which protrudes out of the sand in their natural environment. Previous studies on bivalves have shown greater toxin accumulation in the digestive gland-stomach complex and viscera which may contribute up to 98% of the total toxin, but in some bivalves, a reversal of toxin content from the digestive gland and viscera has been observed, with toxins migrating to alternative tissues over time [48,49]. To fully understand toxin micro-distribution within each species from these experiments, it would be necessary to undertake further fine-scale dissections of each organ as well as feeding for a longer period to allow for toxin exchange between tissues to occur. However, our demonstration of initial toxin accumulation occurring within the digestive glands is useful for the analysis of wild populations, potentially inferring the recency of a contamination event and the occurrence of ongoing toxin uptake.

## 4. Materials and Methods

### 4.1. Paphies australis Collection and Acclimation

Adult *Paphies australis* (*n* = 30) of similar size (ca. 40 mm long) were collected from Delaware Bay (Nelson, New Zealand; 41°09′ S, 173°27′ E) between March and August 2020, chilled (ca. 8 °C) and transported to the laboratory (Cawthron Institute, Nelson, New Zealand) within one hour. The shells were scrubbed and rinsed with sterile seawater to remove biofouling. Control samples (hereafter environmental controls; *n* = 5) were stored frozen (−20 °C) until later TTX analysis to ensure that there was no or only very low TTX in the environmental population. The remaining individuals were placed in aquariums (15-L) which had been thoroughly cleaned and rinsed pre-experiment using detergent and bleach (10%). These were maintained with a recirculating flow of seawater and continuous aeration at 18 ± 1 °C with a 14:10 h light:dark cycle. The bivalves were left to acclimatize for one week and fed the microalgal species *Isochrysis galbana* (2 L, ca. 12 × 10^6^ cells mL^−1^) every second day.

### 4.2. Microencapsulation Method Development

#### 4.2.1. Micro-Algal Encapsulation

To develop the encapsulation method, the dinoflagellate *Alexandrium minutum* (CAWD12, maintained in the Cawthron Institute Culture Collection of Microalgae) was used to determine if microalgal cells could be encapsulated and if the capsules were impermeable. The preparation of agar-gelatin-based microcapsules was modified after Lam et al. [50]. Agar (2% in Milli-Q water; Sigma-Aldrich, St Louis, MO, USA) and gelatin (1% in Milli-Q water; Sigma-Aldrich, St Louis, MO, USA), solutions were mixed in glass beakers using a magnetic stirrer (Labnet International, Inc. AccuPlate^™^, Edison, NJ, USA) at 80 °C until fully dissolved. In a separate beaker, 10 mL of each solution was added and stirred at 1000 rpm (10 min, 60 °C). *Alexandrium minutum* culture (ca. 5000 cells mL^−1^; 10 mL) was added to the agar-gelatin mixture and this mixture was stirred for a further 5 min (1000 rpm, RT). A solution of olive oil (400 mL; extra-virgin) and Span^®^ 80 (2 mL; Sigma-Aldrich, St Louis, MO, USA) was made up separately and the mixture slowly added to the agar-gelatin-algae solution with continuous stirring at 1000 rpm. This was then homogenised (OMNI International, Inc^®^, 1 min) to create an emulsion. The emulsion was left to cool whilst stirring (1000 rpm, 3 h). Following stirring, the solution was transferred into a separating funnel along with 500 mL of sterile seawater to promote separation from the oil causing the microcapsules to aggregate at the bottom layer of the separating funnel. The capsules were collected, washed three times with acetone, filtered through 100 μm and 20 μm filters until all olive oil was removed, rinsed with tap water and then dispersed in seawater.

#### 4.2.2. Tetrodotoxin Encapsulation

The encapsulation method was subsequently trialled using liquid TTX solution (0.7 mg kg^−1^) in lieu of a biological producer. Unfortunately, due to the high solubility of TTX in water, the capsules did not contain any toxin after encapsulation (results not shown). Liquid TTX was then substituted for TTX bound to solid humic acid, a strong chelator, to create a solid substrate to encapsulate. To validate that TTX was binding to the humic acid before adding it to the capsules, a small experiment was undertaken where TTX (10 µL, 6 mg kg^−1^) was diluted in MilliQ-water (1 mL), before adding humic acid (60 mg; Sigma-Aldrich, St Louis, MO, USA) and vortexing (2 min, max. speed). Finally, formic acid (10 µL, >98%; Sigma-Aldrich, St Louis, MO, USA) was added to the solution to investigate the release of TTX from the particles of humic acid and simulate what would happen in the stomach of *P. australis* (pH ~ 4, as measured in this study). Sub-samples of the solution were taken at each step and analysed for TTX concentration (see methods below).

After ensuring that TTX was binding to humic acid, the TTX solution (500 µL; 0.7 mg kg^−1^) was diluted in Milli-Q water (9500 µL) and added to humic acid (500 mg) which was then vortexed (5 min). The protocol described in 4.2.1 was then undertaken using TTX-humic acid solution instead of *A. minutum* cells. Three test individuals of *P. australis* were fed the capsule solution and sacrificed 24 h later. Their digestive glands were dissected before being observed under the microscope. The final concentration of TTX in the capsule solution and its accumulation rate in individual *P. australis* were calculated using the formula:

$$\%\;\mathrm{TTX}\;\mathrm{accumulated}=\;\frac{\mathrm{amount}\;\mathrm{TTX}\left(µg\right)\;\mathrm{in}\;\mathrm{each}\;\mathrm{organ}-\mathrm{amount}\;\mathrm{TTX}\;\left(µg\right)\mathrm{in}\;\mathrm{controls}}{506\;µ\frac{g}{L}\mathrm{TTX}\;\mathrm{given}\;\times \;0.007\;L\;\times \;\mathrm{number}\;\mathrm{of}\;\mathrm{TTX}\;\mathrm{feedings}}\;\times 100$$

#### 4.2.3. Microscopic Characterization of Microcapsules

For the entire method development, microcapsules were microscopically examined using an CKX41 microscope (Olympus, Tokyo, Japan) equipped with a digital camera. Digital images were captured and processed using the cellSens imaging acquisition software (Olympus Life Science, version 1.12, Tokyo, Japan).

### 4.3. Feeding Experiment

Following the acclimation period, *P. australis* of similar size were transferred to individual 1-L glass jars (*n* = 1 per jar) containing 500 mL of filtered seawater with constant aeration. The bivalves (*n* = 20) were individually fed a mixture of concentrated *I. galbana* (93 mL; ca. 12 × 10^12^ cells mL^−1^) and freshly-made TTX capsules (7 mL) five times at days 0, 3, 6, 9 and 12, or were fed *I. galbana* only on the same days for experimental controls. The mixture of TTX and algae instead of TTX capsules only was used to trigger the *P. australis* to open and start filtering their food. A subsample (50 mL) of *I. galbana* culture was collected and analysed for TTX. Five *P. australis* fed TTX microcapsules were harvested on day 7, along with five control individuals. The remaining *P. australis* (five fed the solution of *I. galbana* and TTX capsules and five fed *I. galbana* only) were harvested on day 13 for TTX analysis. Using the weight and TTX concentration from each tissue sample and the total weight of individual *P. australis*, total TTX concentrations in whole organisms were calculated. Faeces samples were harvested from each jar at days 7 and 13 but on closer inspection, we observed that these samples also contained humic acid particles that had aggregated at the bottom of the jar and it was not possible to separate these. No further analysis was undertaken on the faeces as it would not have provided meaningful data. The jars were cleaned and the water was replaced on day 7. At each sampling point, *P. australis* were rinsed with Milli-Q water, left to drain (5 min) and were frozen (−20 °C) until toxin extraction.

#### 4.3.1. Tetrodotoxin Extraction and Analysis Using Liquid Chromatography Tandem-Mass Spectrometry

The TTX extraction protocol was adapted from Biessy et al. (2018). Frozen *P. australis* were shucked, aseptically dissected into two tissue groups: (1) the siphons and digestive gland combined (DGS), and (2) the “rest” mostly composed of the foot, gonads, gills and mantle. Each sample was weighed (ca. 0.3–3 g for the different organs) and placed in a sterile tube (50 mL) containing a corresponding volume (ca. 300–3000 µL) of 1% acetic acid in Milli-Q water. The samples were then homogenised (OMNI International, Inc^®^, Kennesaw, GA, USA; 45 s or until complete homogenisation). Samples were boiled (5 min) and cooled in an ice bath (5 min) followed by brief vortexing. The samples were centrifuged (3200× *g*, 10 min) and 1 mL of the supernatant was transferred to a centrifuge tube (1.7 mL) containing 5 µL of 25% ammonia (Honeywell, Charlotte, NC, USA). The samples were centrifuged again (17,000× *g*, 1 min) and the supernatant was cleaned with the GPC Solid Phase Extraction (SPE) method (Boundy et al., 2015) using Supelclean ENVI-Carb 250 mg/3 mL SPE cartridges (Sigma-Aldrich, St Louis, MO, USA). The extracted tetrodotoxin was analysed and quantified using liquid chromatography tandem-mass spectrometry as described in Turner et al. (2017).

#### 4.3.2. Statistical Analysis

The effect of time, sample type, and their interactions on TTX accumulation were assessed using a mixed effect linear model [51]. This modelling framework addresses the non-independence of the data, considering the individual *P. australis*. “Animal id” was incorporated into the model as a random effect. TTX concentration was log-transformed to linearize the relationship and reduce heteroscedasticity. The log-transformed TTX data were tested for normality with an Anderson–Darling normality test [52]. The marginal means for the combination of the day, sample type and TTX concentrations were estimated and the comparisons among them. Adjusted *p* values for the differences were calculated using the Tukey method. All statistical analyses were performed within the ‘R’ statistical and programming environment [53]. The package *lme4* [54] was used for the mixed effect linear models and the package *emmeans* [55] to estimate the marginal means (least-squares means) for the factor combinations from the mixed-effects linear models.

## 5. Conclusions

A method which involved binding TTX to solid particles of humic acid and encapsulating them in agar-gelatin capsules was successfully developed. This is the first study binding TTX to a solid particle that was then fed to aquatic organisms. This experiment involved a known quantity of TTX-containing microcapsules being fed to *P. australis*. The bivalves only accumulated 0.5 to 1% of the TTX given at each feeding but their TTX concentrations reached of up to 103 µg kg^−1^ after only 13 days, a concentration similar to those found in wild populations and well above the safe threshold recommended by EFSA of 44 µg kg^−1^ in bivalves. This result demonstrated that *P. australis* can accumulate TTX from an external source, thus strengthening the hypothesis that bivalves accumulate TTX from their diet. The method developed in this study will enable TTX dynamics to be explored within different species of bivalves, and other aquatic species. Enhancing knowledge on accumulation and depuration rates in a range of aquatic species will help establish the time needed for safe consumption to occur following the discovery of contamination with TTX, and may provide new insights into why some bivalves accumulate TTX and others do not.

## Figures and Tables

**Table 1:** Tetrodotoxin (TTX) concentrations in solution at various stages in the testing of adsorption and release (under acidic conditions) from humic acid.

Sample	TTX Concentration (ng mL^−1^) in Solution
TTX in water	102.3 ± 5.5
TTX in water + humic acid	29.7 ± 1.8
TTX in water + humic acid + formic acid	82.7 ± 2.1

**Table 2:** Pairwise comparisons (Tukey HSD test) of mean tetrodotoxin concentrations in *Paphies australis* during the feeding experiment, among days, sample type and treatments (CTL = controls fed microalgae only, TTX = organisms fed tetrodotoxin capsules and microalgae, DGS = Digestive gland and siphons pooled together). Bolded values represent statistically significant differences (*p* < 0.05).

			Day 7						Day 13					
			DGS		Rest		Whole		DGS		Rest		Whole	
			CTL	TTX	CTL	TTX	CTL	TTX	CTL	TTX	CTL	TTX	CTL	TTX
Day 7	DGS	CTL		0.022	<0.0001	0.0052								
		TTX			<0.0001	<0.0001								
	Rests	CTL				0.0444								
		TTX												
	Whole	CTL						0.0055						
		TTX												
Day 13	DGS	CTL	0.9999	0.0005	<0.0001	0.0234				<0.0001	<0.0001	<0.0001		
		TTX	<0.0001	0.0131	<0.0001	<0.0001					0.9607	<0.0001		
	Rests	CTL	<0.0001	<0.0001	0.9927	0.0038						<0.0001		
		TTX	0.9998	0.0136	<0.0001	0.001								
	Whole	CTL					0.9998	0.001						<0.0001
		TTX					<0.0001	0.0011						

## Data Availability

Not applicable.
